# Effect of hypothyrodism’s medication (T4) on implant osstointegration: A case series and literature search

**DOI:** 10.1016/j.ijscr.2021.01.040

**Published:** 2021-01-15

**Authors:** Mariam Al-Hindi, Randa Al-Fotawi, Abdulaziz Al-Tamimi, Osama Khalil, Naif Al-Osaimi, Khalid Al-Ghamdi, Khloud Heji

**Affiliations:** a Oral and Maxillofacial Surgery Dept, Dental Collage, King Saud University, Saudi Arabia; b Dental Faculty, King Saud University, Saudi Arabia; c Prosthodontic dept, Dental Faculty, King Saud University, Saudi Arabia

**Keywords:** Dental implants, Hypothyroid disease, Medically compromised patient, T4, Osseointegration

## Abstract

**Introduction:**

Hypothyroidism reduces the recruitment, maturation, and activity of bone cells, decreasing bone resorption and formation. Several investigations have reported that T4 replacement therapy is associated with a significant decrease in bone mineral density in various skeletal parts, while others have failed to corroborate these results. The present study describes both a literature review and our own experience with dental implants in patients with hypothyroidism undergoing T4 replacement therapy.

**Case presentation:**

The study included two parts: a literature review and case series. The literature review included 12 articles documenting the success rate of osseointegrated dental implants in patients with hypothyroidism. The clinical cases were chosen from King Saud University Dental College, Riyadh. The patients’ identity was only available to the main researcher. The inclusion criteria for the clinical cases were: T4-treated hypothyroidism, age 20–60 years, and use of dental implants with a follow-up period of 6–12 months after loading. The exclusion criteria were: any other medical condition alongside hypothyroidism, syndromic hypothyroidism, pregnancy, current smoking, bruxism, and hypothyroidism caused by surgical excision combined with radiotherapy. The following parameters were assessed: insertion torque (IT), crestal bone height (CBH), mesial bone width (MBW), and distal bone width (DBW) at different time points, probing depth, and signs of infection.

**Discussion:**

Seventeen dental implants placed in patients with T4-treated hypothyroidism showed median IT success (42.4 N⋅cm; range: 35–45 N⋅cm). The median crestal bone loss was measured at 6–12 months after loading was 0.6 mm (range: 0.5–0.7. mm). Conversely, the median bone loss differences in MBW and DBW at 6–12 months after loading were 0.3 mm and 0.2 mm, respectively.

**Conclusion:**

After a 1-year follow-up, patients with T4-treated hypothyroidism who had received dental implants fulfilled the criteria for successful implants.

## Highlights

• This is the first case series that study directly the effect of Thyroxin medication on dental implants.
• The presented data would support or defet the data in literature.
• More than one parameter for implant’s success were used for all the presented cases.

## 1 Introduction

Dental implant treatment has transformed oral rehabilitation, vastly improved both fixed and removable treatment choices, and is broadly considered the ideal treatment for tooth loss. Dental implants are surgical components that interface with the jaw or skull to act as an orthodontic anchor or support a dental prosthesis such as a crown, bridge, denture, or facial prosthesis. The success rate of dental implants was 90%–95% in the last 10 years [1]. Systemic conditions can play a negative role in the osseointegration process around titanium implants, especially metabolic bone diseases such as osteoporosis, diabetes mellitus, and hypothyroidism. Bone has two important processes that continue throughout life: osteoblastic bone formation (anabolic activity) and osteoclastic resorption (catabolic activity); as such, bone is considered a highly dynamic, active tissue. The response of bone tissue to assaults such as fracture or implant placement depends on several mechanisms and can be affected by different conditions, so dental surgeons must be aware of the risks and difficulties that can arise before, during, and after implant placement. Moreover, they must know how to manage patients with underlying conditions, especially endocrine disorders [2]. Thyroid disorders predominantly affect women and are the second most common endocrine disease, affecting 1% of the general population [3]. The thyroid gland produces thyroxine hormone (T3) (T4), which regulates carbohydrate, protein, and lipid metabolism; thyroxine is also essential for normal bone turnover and increases the action of other hormones, such as catecholamines and growth hormone [2]. The relationship between thyroid diseases and bone metabolism was first described 100 years ago. In the 1970s, it became apparent that thyroid-associated metabolic bone disease had specific histological features. Hyperthyroidism and hypothyroidism are caused by impairment of the anterior pituitary gland or thyroid gland [2]. Hypothyroidism reduces the recruitment, maturation, and activity of bone cells, which decreases bone resorption and formation [3]. Thyroid disorders and thyroid hormone medications also influence bone metabolism [4]. Studies evaluating bone mineral density (BMD) changes in patients with hypothyroidism have indicated that T4 replacement therapy is associated with a significant decrease in BMD in various skeletal parts, while several other studies have failed to corroborate these results. In the present study, a literature search identified five clinical case series involving patients with T4-treated hypothyroidism who were given osseointegrated titanium dental implants. The treatment was performed in King Saud University clinic with patient consent; the identity of the patients was only revealed to the main author. All patients underwent surgery conducted by the same experienced surgeon. To our knowledge, the present case series was the first to directly study the effect of thyroxin medication on dental implants by assessing the parameters of successful dental implants.

## 2 Literature search and case presentation

The study comprised two parts: a literature review and a documentation of five patients with hypothyroidism who received dental implants.

The literature review included the PubMed, Google Scholar, and NCBI databases and considered articles published between 1999 and 2019. Articles that were deemed published with low impact or unrelated to the topic were excluded. The electronic search was supplemented by manual searches of the reference lists from the selected full articles. Another manual search was applied to peer-reviewed English language journals, focusing on articles related to the effect of controlled hypothyroidism on dental implant treatment outcomes. Studies were required to meet the inclusion criteria (listed below) and to have sufficient information in their title and abstract to allow a clear decision to be made. All studies that met the inclusion criteria underwent a validity assessment.

**Fig. 1:**
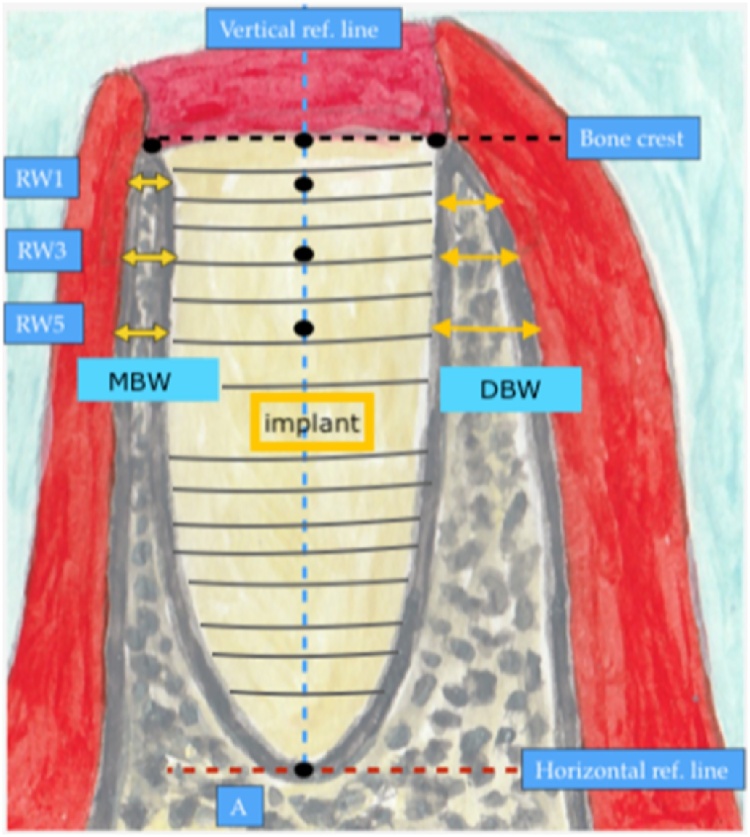
The illustration shows how the bone height and width were measured immediately (D0) and 1 year (D1) after implant insertion. Two reference lines are drawn: the blue, vertical, broken line denotes the center of the implant crossing the apical reference point, while the red, horizontal, broken line. Present the apical limit for measuring the crestal bone height. The mesial bone width (MBW) and distal bone width (DBW) are also shown.

The inclusion criteria for patients were (1) receipt of dental implants, (2) diagnosis of hypothyroidism, and (3) treatment using T4 replacement. The exclusion criteria were (1) multiple systemic diseases, (2) poor oral health, (3) risk factors such as smoking, bruxism, etc. Successful implant after surgery was defined based on (1) clinically stable implant immediately after insertion; (2) implant insertional torque (IT) of > 32 N⋅cm recorded immediately after insertion to assess primary stability; (3) absence of inflammation, including redness, hotness, swelling, pain, and loss of function; (4) bone loss of < 2 mm in the coronal portion after 1 year, detected radiographically. Fig. 1 shows a detailed illustration of the how the bone height and width were measured immediately after implant insertion and at the 1 year follow-up (Fig. 1). Information regarding age, sex, bone loss around the implant, and infection around the implant were recorded for later use.

Written informed consent was obtained from the patient for publication of this case report and the accompanying images. This work has been reported in line with the SCARE 2018 criteria [5].

### 2.1 Literature review

The literature review included 13 articles relating to hypothyroidism and osseointegration of dental implants in the last 20 years (1999–2019). In 2001, Attard et al. investigated prosthodontic management in medically treated patients with hypothyroidism; they concluded that patients with hypothyroidism had more soft tissue complications and more bone loss in the 1st year of loading than their matched controls. Additionally, the implant survival outcomes did not influence the success of the dental implant [6]. In 2002, Elsubeihi et al. conducted research on implants in medically challenged patients [3]; 27 patients with 82 implants were compared with a control group of 81 implants. The results showed more bone loss in the 1st year in patients with hypothyroidism. In subsequent years, bone loss decreased to the same level as the control group. Overall, patients with hypothyroidism showed no higher risk than patients without disease with regards to the success rate of implants [2]. In the same year, Attard et al. compared dental implants in patients with hypothyroidism with those in a control group of 29 patients. That study showed that patients with hypothyroidism had more soft tissue problems and bone loss than the control group in the 1st year, but no noticeable difference in the subsequent years [4]. In 2008, Alsaadi et al. investigated the impact of local and systemic factors on the incidence of late oral implant loss. In that study, 387 patients with hypothyroidism had 1403 dental implants placed, and the success rate was comparable to that of medically fit patients. They concluded that hypothyroidism does not influence dental implant success [7]. In 2011, Zahid et al. studied the effects of thyroid hormone abnormalities on periodontal disease status, concluding that periodontal therapies, including dental implant placement, are safe and confer no increased risk of treatment failure as long as the thyroid status is controlled [8]. In 2013, Diz et al. assessed dental implants in medically compromised patients and concluded that peri-implantitis or peri-implant conditions were insufficient and even conflicting for the majority of compromising systemic aspects [9]. They suggested that future studies should investigate peri-implant tissue health and maintenance in compromised patients. In 2015, Sehravat tested effect [2]. In 2017, Dalago et al. investigated the risk indicators of peri-implantitis. In that study, 769 implants were placed in patients with thyroid disease, and no significance difference in bone loss after loading was detected [10]. In the same year, Guilherme et al. studied the survival/success of dental implants with acid-etched surfaces [11]. They conducted a retrospective evaluation after 8–10 years in four patients with hypothyroidism; one had developed peri-implantitis and the authors concluded that implants with acid-etched surfaces showed high survival and success rates after 8–10 years [11]. In 2018, Pranjali et al. conducted a retrospective study to assess the survival rates of dental implants in medically compromised patients. The study involved a group of 35 patients with diabetes, 20 with osteoporosis, 10 with hypothyroidism, and 25 with cardiovascular disease (CVD). The implants had a 76% survival rate, whereas in a control group of 111 patients, the survival rate was 90% [12].

In the same year, Jafar et al. investigated whether vitamin D level was associated with the success rate of dental implants in females with hypothyroidism. That study involved a 3-year follow-up and concluded that females who took thyroid therapy were more likely to show implant failure than other females [13]. Females with hypothyroidism are susceptible to soft tissue complications in the first stage after the first surgery. In 2020, Anuj et al. concluded that, among medically compromised conditions, a higher failure rate was found in diabetes patients. On the same study, they reported 25 patients were diabetes, 16 were having osteoporosis, 10 with organ transplant, 10 with hypothyroidism, and five with CVDs, the author concluded that among medically compromised conditions, a higher failure rate was found in diabetes [14].

The purpose of the present study was to (1) investigate the effect of thyroxin T4 medication on the osseointegration of dental implants, and (2) report five clinical cases.

## 3 Case reports of patients with hypothyroidism who received dental implants

### 3.1 First case

**Fig. 2:**
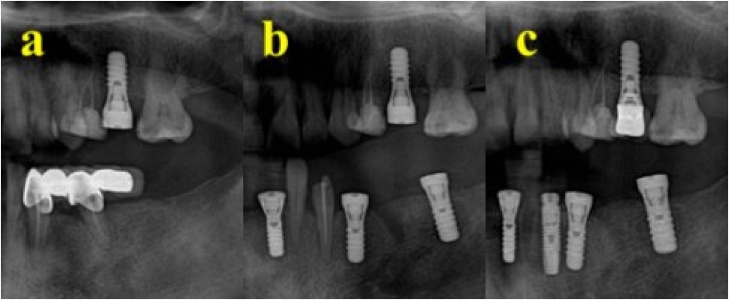
Case one showing (A) inserted upper implant, (B) inserted lower implants, (C) loading of the upper implant 12 months after insertion.

A 44-year-old female patient attended for replacement of her lower right fixed partial denture using dental implants and tooth No. 25. She reported having hypothyroidism and was taking 75 mg of thyroxine daily. She reported no remarkable family, genetic, psychological, or social history. The patient’s BMI was 35 and she had no other medical conditions. She was examined by a prosthodontist and a treatment plan was prepared. On the day of dental implant insertion, a radiograph was taken to assess bone level before implant bed preparation (Fig. 2). A 13-mm bone level was measured from the inferior alveolar nerve canal to the crestal bone at teeth Nos. 44 and 46. The bone level was 12 mm at tooth No. 25. The implant bed was prepared for two implants under local anesthesia using 2% lidocaine. Next, an incision was made in the crest of the ridge, an envelop flap was raised buccally and lingually, and the implant bed was prepared. Bone level dental implants measuring 4.1 × 10 mm (Straumann® Dental Implant System) were used. Initial implant stability was achieved, and the mean initial torque measurement was 40 N⋅cm. Clinical examination showed no signs of bone dehiscence buccally or lingually, and the patient tolerated the procedure very well. The patient was instructed not to eat or chew on the operated side for the first 12 weeks after surgery. The implant was loaded 3 months after insertion. The patient was referred for re-evaluation after 1 year, while her clinical evaluation was conducted along with orthopantomography. The implants replacing teeth Nos. 25, 44, and 46 were stable and showed no signs of inflammation. No bone loss was detected on radiography. During the year, the patient received several implants that were crowned later. The patient was satisfied with the treatment and discharged 1 year after loading.

### 3.2 Second case

**Fig. 3:**
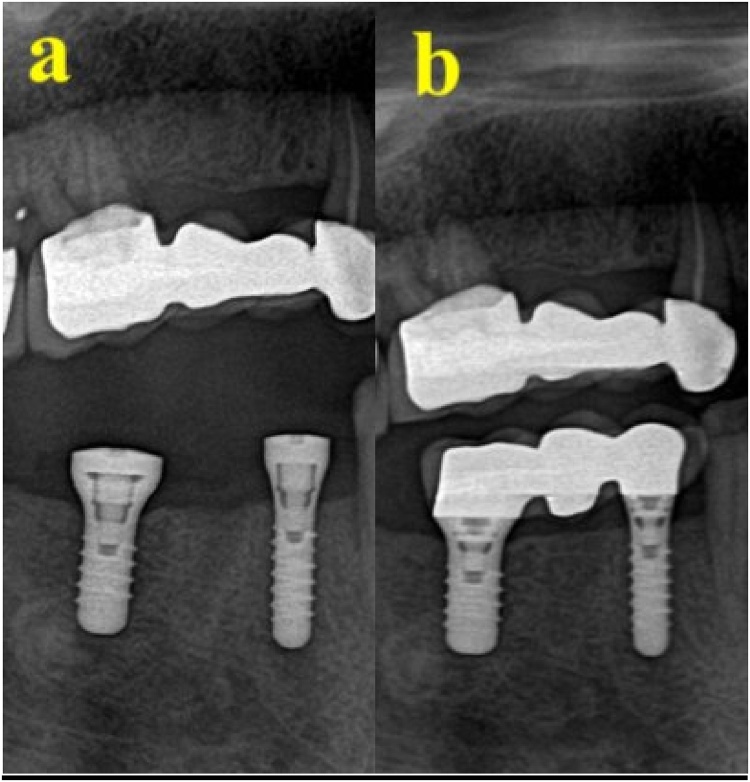
(A) Two implants inserted in the premolar-molar area before loading. (B) Implants are loaded with a bridge, replacing the premolar area.

**Fig. 4:**
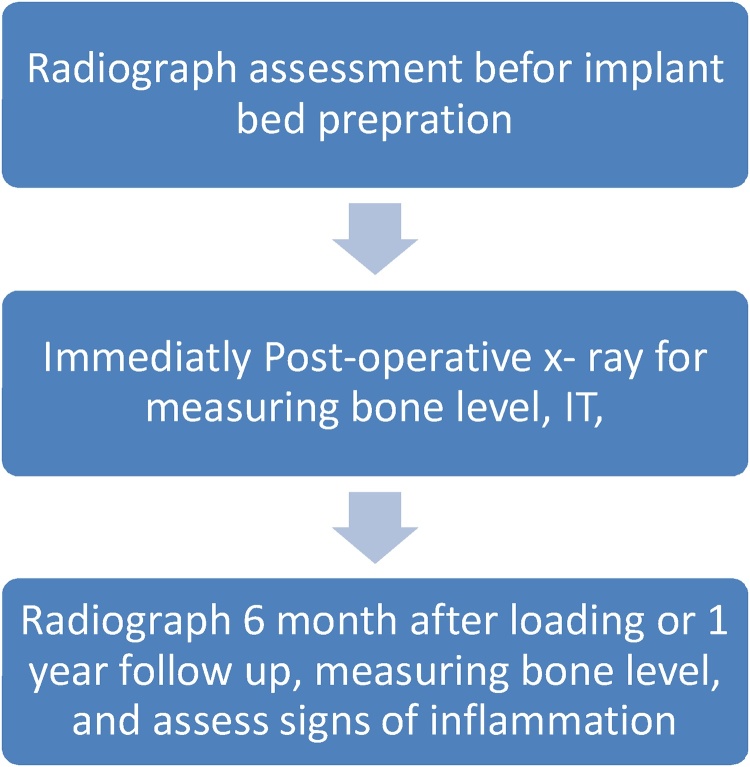
Timeline for assessment of implant and radiographs.

A 39-year-old female patient attended for replacement of an extracted tooth (No. 46). She reported having hypothyroidism and was taking regular thyroxine (75 mg during the week and 100 mg during the weekend). She reported no remarkable family, genetic, psychological, or social history. The patient’s BMI was 42 and she had no other medical conditions. The patient was seen by a prosthodontist to arrange a treatment plan. Radiographs were taken to assess the bone level before implant bed preparation (Fig. 3). The implant bed was prepared under local anesthesia using 2% lidocaine. Next, an incision was made in the crest of the ridge, an envelop flap was raised buccally and lingually, and the implant bed was prepared. A bone level dental implant measuring 4.1 × 10 mm (Straumann® Dental Implant System) was used. The patient tolerated the procedure well, and clinical examination showed no signs of bone dehiscence buccally or lingually. Initial implant stability was achieved, with a torque of 45 N⋅cm. The patient was instructed not to eat or chew on the operated side for the first 12 weeks after surgery. The patient reported an uneventful recovery period with no complications. An implant was inserted in tooth No. 46 and was loaded after 12 weeks in a satisfactory condition. The patient was recalled after the examination and showed a stable implant under clinical examination, with no signs of inflammation and no crestal bone loss in the periapical radiographic films (Fig. 4). The patient was satisfied with the treatment and was discharged 1 year after loading (Table 1).

**Table 1:** Summaries the data taken from the clinical cases including number of implants, Initial torque, Bone height and The probing Depth.

Case no.	no of implants	IT	Bone parameters D0			Bone parameters D1			Signs of infections
			H	MBW	DBW	H	MBW	DBW	Probing depth (mm)
1	7	45	10.5	3.5	4.5	10	3	4.3	3
2	1	35	10	2.5.	4.2	9.4	2.5.	3.8	2
3	3	42	10.5	4	3.8	9.8	3.2.	3.6	2
4	3	44	11	3.5.	3.2	10.4	3.5.	3.0	2
5	2	45	10.5	4.5.	2.5	10	4.4.	2.4	3
Median		**42.2**	**10.5**	**3.6.**	**3.6**	**9.9**	**3.3.**	**3.4**	2.4

IT: Insertion Torque, D0: Day 0, D1:12 month after insertion, H: horizontal, MBW: Mean bone width, DBW: Distal bone width.

### 3.3 Third case

**Fig. 5:**
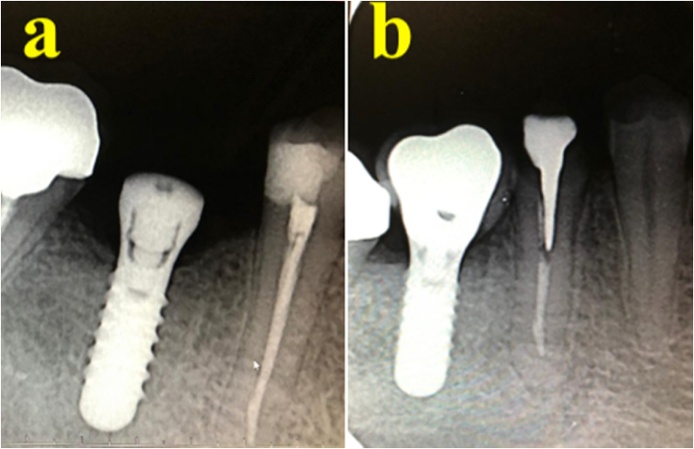
(A) Single implant inserted to replace molar tooth. (B) 12 months after loading of the implant.

A 20-year-old female patient attended to replace extracted teeth Nos. 44 and 46. The patient reported having hypothyroidism and was taking thyroxine (100 mg daily). She also reported no remarkable family, genetic, psychological, or social history. The patient’s BMI was 30 and she had no other medical conditions. She had been undergoing orthodontic treatment for the previous 3 years. The patient was evaluated by a prosthodontist. A radiograph was taken to assess bone level before implant bed preparation. The implant bed was prepared under local anesthesia using 2% lidocaine. Next, an incision was made in the crest of the ridge, an envelop flap was raised buccally and lingually, and the implant bed was prepared. Bone level dental implants measuring 4.1 × 10 mm and 4.1 × 8 mm (Straumann® Dental Implant System) were used to replace teeth Nos. 44 and 46, respectively. The implants were inserted in teeth Nos. 44 and 46, with an initial torque of 35 N⋅cm. The patient was instructed not to eat or chew on the operated side for the first 12 weeks after surgery. The patient reported an uneventful recovery period with no complications and tolerated the procedure very well. Crowning was completed after 12 weeks. The patient was called after 1 year for evaluation. Clinical examination showed stable implants that allowed signs of inflammation and crestal bone loss to be assessed (Fig. 5). Crestal bone loss of 1 mm occurred around the area of tooth No. 46. The patient was satisfied with the treatment and was discharged 1 year after loading.

### 3.4 Fourth case

**Fig. 6:**
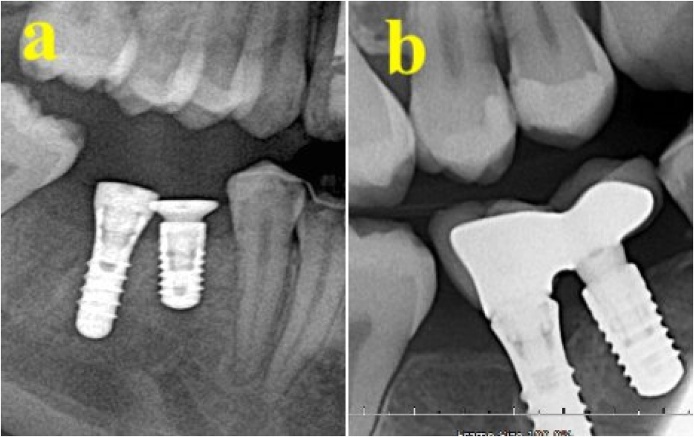
(A) Two implants after insertion, with healing cap. (B) 12 months after loading of the two implants.

A 42-year-old female patient presented for replacement of her extracted 16, 45, and 46. The patient had a history of hypothyroidism and was taking thyroxine (60 mg daily). She reported no remarkable family, genetic, psychological, or social history. The patient’s BMI was 45 and she had no other medical conditions. The patient was evaluated by a prosthodontist, and radiographs were taken to assess bone level before implant bed preparation. The implant bed was prepared under local anesthesia using 2% lidocaine. Next, an incision was made in the crest of the ridge, an envelop flap was raised buccally and lingually, and the implant bed was prepared. Bone level dental implants measuring 4.1 × 10 mm (Straumann® Dental Implant System) were used. The implants were inserted at teeth Nos. 16, 45, and 46 in two separate sessions. The patient was instructed not to eat or chew on the operated side for the first 12 weeks after surgery. The patient reported an uneventful recovery period with no complications and tolerated the procedure very well. The patient received her crown after 10 weeks. She was referred for review after 1 year. Clinical examination revealed a stable implant with no signs of inflammation. The torque of implant No. 16 was 40 N⋅cm, while that at Nos. 45 and 46 was 35 N⋅cm. No crestal bone loss was detected (Fig. 6). The patient was satisfied with the treatment and discharged 1 year after loading.

### 3.5 Fifth case

**Fig. 7:**
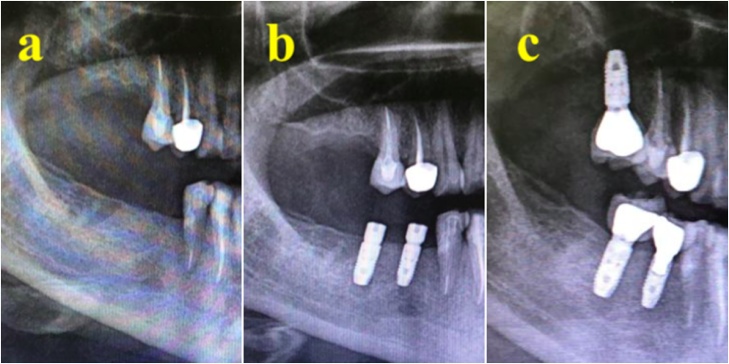
(A) Area after extraction. (B) Two implants replacing lower premolar and molar. (C) 12 month after implant loading.

**Fig. 8:**
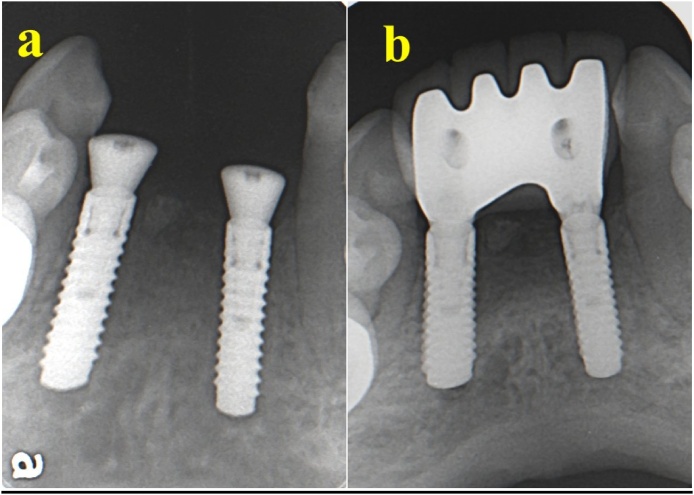
(A) Two implants inserted to replace missing lower anterior teeth. (B) 12 months after loading.

A 47-year-old female patient attended to replace extracted teeth Nos. 32, 31, 41, and 42. The patient reported having hypothyroidism and was taking thyroxine (75 mg daily). She also reported no remarkable family, genetic, psychological, or social history. The patient’s BMI was 40 and she had no other medical conditions. The patient was evaluated by a prosthodontist. A radiograph was taken to assess bone level before implant bed preparation. Implant beds were prepared for two implants under local anesthesia using 2% lidocaine. Next, an incision was made in the crest of the ridge, an envelop flap was raised buccally and lingually, and the implant bed was prepared. A bone level dental implant measuring 3.3 × 12 mm (Straumann® Dental Implant System) was used. Implants were inserted in teeth Nos. 32 and 42, with an initial torque of 35 N⋅cm. The patient was instructed not to eat or chew on the operated side for the first 12 weeks after surgery. The patient reported an uneventful recovery period with no complications and tolerated the procedure very well. Crowning was completed after 14 weeks. The patient was called after 1 year for evaluation. Clinical examination revealed a stable implant with no signs of inflammation. The torque of the implant at No. 32 was 30 N⋅cm, while that of the implant at No. 42 was 35 N⋅cm. No crestal bone loss was detected (Fig. 7, Fig. 8). The patient reported satisfaction with the treatment and was discharged 1 year after loading.

## 4 Discussion

Thyroid disorders are the second most common endocrine disease, affecting 1% of the general population and predominating in women [4]. The relationship between thyroid diseases and bone metabolism was first described 100 years ago. Hypothyroidism reduces the recruitment, maturation, and activity of bone cells, which decreases bone resorption and formation [3]. Therefore, BMD is a major concern when patients on medication for hypothyroidism require dental implant placement, because replacement therapy using T4 is associated with a significant decrease in BMD in various skeletal parts [4]. One examination of dental clinical studies involving patients with hypothyroidism revealed that this disease had no effect on implant success. These clinical cases suggested that hypothyroid medication alone does not affect implant success. Although the author of the report did not directly measure bone density in all reported cases, the IT, which is correlated with bone density, was reported in all implants [15].

The criteria for successful dental implants in hypothyroidism have been investigated extensively in the literature, but are still debated by researchers. In 2001, the success of implant osseointegration was checked during the 2nd stage of surgery and every year after using clinical and periapical radiography to assess marginal bone loss [6]. A 2002 analysis of the marginal bone by Elsubeihi et al. was the considered the main factor for implant success; however, the method of analysis was not mentioned [3]. In the same year, Attard et al. followed the same parameters as Nikolai in assessing the implant’s success [4]. In 2008, Al-Saadi et al. identified implant failure if radiolucency was detected around the implant in a periapical radiograph [7]. Dalago et al. [10] reported their criteria for successful implants, such as the probing depth (1.5–2 mm) and bleeding upon probing [10]. This was followed by a study by Nicoli et al. [11,16], who concluded that absence of peri-implantitis and radiolucency around implants was the deciding factor of success [15].

Other factors have been considered to assess the success of dental implants, such as IT, which reflects bone density and volume [16]. Specifically, an average IT of 35 N cm is associated with implant success [16].

Chai et al. [17] found that the IT is significantly correlated with implant site bone density [17] and that it may be a viable and practical means to assess mandibular bone quality in patients with compromised general bone density. Another study by Venkatakrishnan et al. [18] found that, in osteoporotic bone, the amount of stress–strain given at 32 N equal to 40 N, were considered sufficient for implant success [18]. The correlation between IT and bone quality was also investigated by Makary et al. [19]. They found that, for bone types I–IV, the initial torque achieved was 15–150 N cm, with a mean of 75 N cm [19]. In the present study, the range of reported torque was 35–45 at 42 N cm, which may indicate primary success of implant but more importantly to the quality of bone.

Conversely, there is no consensus regarding the effects of IT above 50 N cm. Duyck et al. [20] concluded that > 50 N cm IT would lead to a high bone loss rate around implants [20], whereas Greenstein et al. 2017 found that high IT (≥ 50 N cm) reduced micromotion and did not damage bone [21].

In 2020, Parihar et al. suggested that measuring bone loss is crucial to implant success using periapical radiographs [14]. They stated that bone loss of more than 1 mm in the 1st year and of 0.3 mm in each subsequent year are considered failures. The authors in the present study measured marginal bone loss 1 year, or at least 6 months, after loading, which was reported at 0.6 mm.

Assessment of peri-implant marginal bone level (PIMBL) is an integral part of implant evaluation [14]. Preserving the PIMBL is paramount to implant success. Two important parameters are related to adequate bone quality and quantity: osseointegration and contour of the overlying soft tissues. Importantly, PIMBL is a multifactorial issue; it depends on surgical technique single vs. two-stage surgery, the loading time, and the grafted socket. In addition to the implant, tooth to implant distance, implant to abutment interface, which can be either supra-or subcrestal placement of the implant [22]. In the literature, this subject has been investigated extensively. In one study, there was no significant difference in survival rate or PIMBL between two-stage and one-stage surgery when the ITI®, Branemark®, and Astra Tech Implant® systems were used [[23], [24], [25]].

Inflammation of tissues surrounding the implants can affect the mucosa and bone, which is called peri-implantitis [26]. If only the soft mucosa is involved, the inflammation is known as peri-implant mucositis. This inflammatory process can be detected using periodontal probing to identify bleeding and/or suppuration. Meanwhile, bone loss is assessed using radiography. Importantly, even without signs of inflammation, subjects may experience some bone loss as a part of natural bone remodeling early after implant installation and loading.

The survival rate of dental implants placed in patients with hypothyroidism indicates a high success rate for the placement of dental implants [27]. In the present study, implants were placed in five patients with hypothyroidism. After a 1-year follow-up at King Saud University, the results were encouraging, as all patients fulfilled the criteria for successful implants.

## 5 Conclusion

Dental implants placed in patients with hypothyroidism fulfilled the criteria for successful implantation in the present case series. Further documentation is required under several different clinical situations to conﬁrm (or falsify) the ﬁndings in this report.

## Declaration of Competing Interest

The authors report no declarations of interest.

## Funding

No fund for this paper.

## Ethical approval

The present case report was obtained from IRB Approval of Research Project No. E-20-4857, reference No. E-19-4356/IRB.

## Consent

Written informed consent was obtained from the patient for publication of this case report and accompanying images. A copy of the written consent is available for review by the Editor-in-Chief of this journal on request.

## Registration of research studies

Research not registered.

The novel aspects and/or learning points:

1. This the first case series which directly study the effect of Thyroxine medication and presented with successful dental implants at 6–12 months after loading.
2. RESULTS will add to the fund of knowledge in literature supporting the success of dental implant in those category of patient.

## Guarantor

Randa Alfotawi.

## Provenance and peer review

Not commissioned, externally peer-reviewed.

## CRediT authorship contribution statement

**Mariam Al-Hindi:** Supervision, Writing - original draft, Methodology. **Randa Al-Fotawi:** Writing - review & editing, Validation. **Abdulaziz Al-Tamimi:** Data curation. **Osama Khalil:** Data curation. **Naif Al-Osaimi:** Data curation. **Khalid Al-Ghamdi:** Visualization. **Khloud Heji:** Methodology, Visualization, Formal analysis.
